# Respiratory and Ocular Symptoms Among Employees of an Indoor Waterpark Resort — Ohio, 2016

**DOI:** 10.15585/mmwr.mm6637a5

**Published:** 2017-09-22

**Authors:** Sophia K. Chiu, Nancy C. Burton, Kevin H. Dunn, Marie A. de Perio

**Affiliations:** ^1^Epidemic Intelligence Service, CDC; ^2^Division of Surveillance, Hazard Evaluations, and Field Studies, National Institute for Occupational Safety and Health, CDC; ^3^Division of Applied Research and Technology, National Institute for Occupational Safety and Health, CDC.

In July 2015, a municipal health department in Ohio received complaints of respiratory and ocular symptoms from patrons of an indoor waterpark resort. In response, the health department conducted an online survey in August 2015 through which 19 (68%) patron and employee respondents reported eye burning, nose irritation, difficulty breathing, and vomiting. On August 11, 2015, the health department requested a health hazard evaluation by CDC’s National Institute for Occupational Safety and Health to characterize the prevalence of symptoms among employees and determine the etiology of work-related symptoms. In January 2016, CDC investigators performed a cross-sectional epidemiologic study, environmental sampling, and ventilation system assessment (*1*). Findings suggested that chlorine disinfection byproducts and environmental conditions contributed to a higher prevalence of work-related respiratory and ocular symptoms among employees in the waterpark compared with employees in other resort areas. Recommendations included servicing the ventilation system, changing work practices to decrease the amount of disinfection byproduct precursors, and responding promptly to employee reports of symptoms.

Indoor waterparks are enclosed recreational environments that can be associated with illness caused by endotoxins and disinfection byproducts. Chlorine disinfection byproducts such as chloroform and chloramines are formed when chlorine, the most commonly used disinfectant in aquatic venues (e.g., pools), reacts with other chemicals in the water. For example, chloramines form when chlorine combines with nitrogen-containing substances, such as urine, sweat, skin cells, and personal-care products from swimmers’ bodies (*2*). Levels of disinfection byproducts in aquatic venues and surrounding air depend on factors such as water chemistry, bather load and hygiene, amount of splashing and spraying (i.e., disturbance of water surface), and ventilation (*3*). Disinfection byproducts can lead to water and air quality issues, particularly in indoor aquatic facilities, and can cause ocular and respiratory irritation.

## Epidemiologic Investigation

As part of a 3-day site visit in January 2016, CDC investigators administered a questionnaire to resort employees concerning demographics, work and medical history, and specific work-related symptoms occurring during the preceding 4 weeks. Symptoms were considered work-related if they started at work and improved when away from work. Participants were asked to exclude symptoms associated with a cold or respiratory infection. Employees aged ≥18 years provided oral informed consent. Written informed consent for participation was obtained from parents or legal guardians of employees aged <18 years.

Resort employees in the aquatics department and the waterpark concession stand were classified as waterpark employees (exposed); employees working in other areas of the resort were classified as nonwaterpark employees (unexposed). The frequency of work-related symptoms was assessed. A case was defined as three or more work-related symptoms (eye irritation, nose irritation, cough, wheezing, shortness of breath, chest tightness, or sore throat) of any duration occurring in a resort employee during the preceding 4 weeks. An adjusted prevalence ratio and 95% confidence interval was calculated using log-binomial regression to assess variables associated with meeting the case definition.

Among 112 employees working at the resort during the site visit, 91 (81%) participated. Median age was 19 years (range = 15–65 years), and 47 (52%) were male. Forty-eight (53%) employees reported any work-related symptom, among whom 12 (25%) had taken a median of 2 days off work (range = 1–5), and seven (15%) sought medical care for work-related symptoms in the preceding 4 weeks. The most frequently reported work-related symptoms among waterpark employees were eye irritation (62%), cough (56%), and nose irritation (51%) (Table).

**TABLE:** Work-related symptoms* reported by waterpark and nonwaterpark employees in the preceding 4 weeks — indoor waterpark resort, Ohio, January 2016

Symptom	No. (%)	
	Waterpark employees**^†^** (n = 45)	Nonwaterpark employees**^§^** (n = 46)
Any symptom	37 (82)	11 (24)
Met case definition^¶^	24 (53)	5 (11)
Eye irritation	28 (62)	6 (13)
Cough	25 (56)	3 (7)
Nose irritation	23 (51)	3 (7)
Wheezing	19 (42)	2 (4)
Shortness of breath	14 (31)	3 (7)
Chest tightness	14 (31)	3 (7)
Sore throat	4 (9)	3 (7)

* Began while at work and improved away from work; not associated with a cold or respiratory infection.

^†^ Employees in the aquatics department and the concession stand contained within the waterpark.

^§^ Employees in the other resort areas (hotel front desk, office, arcade, gift shop, and bar) or departments (housekeeping, security, and maintenance).

^¶^ Reported three or more work-related symptoms.

Twenty-nine (32%) employees met the case definition, 24 (83%) of whom were waterpark employees and five (17%) were nonwaterpark employees. Being a waterpark employee and having current asthma were associated with meeting the case definition, but age <18 years, male sex, and being a current smoker were not. After adjusting for age as a continuous variable and current asthma, waterpark employees were more likely to meet the case definition than were nonwaterpark employees (adjusted prevalence ratio = 3.8; 95% confidence interval =1.4–16.2).

## Environmental and Ventilation Investigation

In this facility, water features included a children’s play area, activity pool, rain fortress with a splash area and bucket periodically dumping 1,000 gallons of water, four waterslides, and a hot tub and spa. The resort also included a hotel, conference center, bar, gift shop, arcade, concession stand, and office area. The same company had been operating the resort since 2013. Area air samples for endotoxins, chlorine, and chloroform collected on 3 consecutive sampling days at six waterpark locations detected levels that were well below occupational exposure limits (*1*). Air temperature and relative humidity in the waterpark were logged each minute while the waterpark was open over 3 sampling days. Daily average air temperature was below and relative humidity was above the range recommended for aquatic environments (*4*).

Water chemistry tests were performed using a standard color-matching test kit at four waterpark locations. Concentrations of combined chlorine, of which chloramines are a subset, in the water were at or above the waterpark’s internal standard of 0.2 ppm on all 3 days of the evaluation, indicating the presence of chloramines. No *Legionella* or mycobacteria were cultured from water samples from the hot tub and spa.

Assessment of the heating, ventilation, and air-conditioning (HVAC) system identified multiple areas of concern. According to blueprints, the HVAC system design should be able to meet current standards and guidelines in CDC’s Model Aquatic Health Code (*5*). However, on visual inspection, the fans of five of the waterpark’s six HVAC units were not operational, substantially reducing airflow in the waterpark. The waterpark air distribution system did not provide an airflow pattern with sufficient air movement just above the water surface and deck (where volatilized disinfection byproducts, which are heavier than air, accumulate) to direct contaminated air toward air return intakes. The return air was partially recirculated and the rest was exhausted out of the waterpark through stacks on the roof.

## Recommendations

Recommendations based on the hierarchy of controls approach were provided to the resort.* Engineering controls such as maintenance and repair of the waterpark’s HVAC systems and possible reconfiguration of the air distribution system to improve removal of air contaminants just above the water surface and deck were advised, as was encouraging patrons and aquatics department employees to shower before entering the water to reduce the amount of disinfection byproduct precursors (e.g., urine, sweat, skin cells, and personal-care products) that swimmers introduce into the water. CDC also recommends that swimmers take regular bathroom breaks. Other recommendations included encouraging prompt reporting of symptoms by employees to their supervisors and implementation of a system to track and follow up on reports by resort management to identify possible causes and take appropriate corrective actions.

## Discussion

Although airborne concentrations of chlorine and chloroform in the aquatic resort were low, a constellation of work-related symptoms consistent with disinfection byproduct exposure was approximately four times more common among waterpark employees than among nonwaterpark employees. Similar respiratory and ocular symptoms have been described in outbreaks at indoor aquatic venues implicating disinfection byproducts (*6*–*8*). Water chemistry tests indicated the presence of combined chlorine, including chloramines. HVAC systems, which play an important role in removing air contaminants, were poorly maintained and not operating properly. This was reflected by air temperatures below and relative humidity above recommended ranges. Endotoxin levels were low, and neither *Legionella* nor mycobacteria was detected during sampling, suggesting that these known causes of respiratory and ocular symptoms associated with aquatic facilities were less likely to have contributed to symptoms at this indoor waterpark than disinfection byproducts. Together, investigation findings suggest that disinfection byproducts and environmental conditions likely contributed to the higher prevalence of symptoms among waterpark employees.

The findings in this report are subject to at least three limitations. First, the evaluation occurred in the winter, a period of potentially lower exposure because the waterpark was open for fewer hours. However, this would likely result in underestimation of an effect. Second, personal air samplers for disinfection byproducts or endotoxins could not be placed on waterpark employees because they could interfere with job duties or get wet and malfunction. This limited the ability to evaluate associations between exposures and symptoms at the individual employee level. Finally, disinfection byproducts are a large class of compounds, but air levels of only one representative member, chloroform, were assessed. Chloramines have been previously associated with irritation symptoms like those reported in this facility (*9*); however, no reliable analytic method to measure them in air or water currently exists (*10*).

Indoor waterparks constitute an expanding industry. The first indoor waterpark resort in the United States opened in 1994. By 2015 there were an estimated 192 facilities nationwide, attracting millions of visitors each year. This investigation highlights the need for vigilant monitoring and maintenance of ventilation and water systems to prevent illness in these large, complex indoor aquatic facilities and for public health officials, clinicians, and operators of indoor waterparks to understand the risk for respiratory and ocular symptoms in patrons and employees.

**Summary**

**What is already known about this topic?**

Indoor waterparks are complex environments where problems with air and water quality can result in illness. Chloramines, formed when disinfectant chlorine reacts with nitrogen-containing substances (e.g., urine, sweat) from swimmers’ bodies, are known causes of ocular and upper respiratory symptoms in aquatic facilities.

**What is added by this report?**

Investigation of reported illness in an indoor waterpark resort in Ohio found that waterpark employees were approximately four times more likely to have work-related ocular and respiratory symptoms than were employees in other resort areas. Environmental assessment found that levels of combined chlorine, of which chloramines are a subset, in water exceeded recommended guidelines, but levels of chlorine and chloroform (a representative disinfection byproduct) in air were low. Improperly functioning ventilation systems, resulting in accumulation of disinfection byproducts and temperature below and relative humidity above recommended ranges, likely contributed to the higher prevalence of symptoms among waterpark employees compared with nonwaterpark employees.

**What are the implications for public health practice?**

To prevent recreational water–associated illness caused by endotoxins and disinfection byproducts in indoor waterparks, vigilant monitoring and maintenance of ventilation and water systems are needed. Employees and patrons of indoor waterparks should promptly report symptoms, which might indicate that further attention to water and air quality and ventilation system functioning is needed. Showering before entering the water and taking regular bathroom breaks can reduce levels of disinfection byproduct precursors introduced into the water.

## Footnotes

* The hierarchy of controls is a framework that groups actions by their likely effectiveness in reducing or removing hazards from the workplace. Levels in the hierarchy include elimination, substitution, engineering controls, administrative or work-practice controls, and personal protective equipment. Additional information on the hierarchy of controls is available at https://www.cdc.gov/niosh/topics/hierarchy/.
